# Wee1 and Chk1 – crosstalk between key players in replicative stress

**DOI:** 10.18632/genesandcancer.61

**Published:** 2015-05

**Authors:** Priyanka Saini, Yizhu Li, Matthias Dobbelstein

**Affiliations:** Institute of Molecular Oncology, Ernst Caspari Haus, Göttingen Center of Molecular Biosciences, Faculty of Medicine, University of Göttingen, Göttingen, Germany

Replicative stress is a tumor cell-associated feature that includes the accumulation of stalled or collapsed replication forks. With the DNA polymerases lagging behind the helicases, these structures contain extended regions of single stranded DNA, leading to the activation of a damage signaling pathway that includes the kinases Ataxia Telangiectasia Mutated-Related (ATR) and Chk1. The specific increase of replicative stress in tumor cells raises the perspective of further enhancing this stress condition for therapeutic purposes, ultimately resulting in cancer cell death [1]. Targeting the replicative machinery for DNA can be achieved by established chemotherapeutic drugs, such as nucleoside analogues, platinum compounds, or topoisomerase inhibitors. This can be viewed as one example of the more general concept of exploiting tumor-supportive molecular machineries as drug targets [2].

On top of interfering with DNA replication as such, targeting signaling pathways to enhance replicative stress has turned out as a viable option with clinical perspectives. In particular, interfering with ATR-Chk1 signaling promotes the death of proliferating cancer cells [3], and inhibitors of both kinases are currently evaluated in clinical trials. In addition, however, the kinase Wee1 turned out as an at least similarly promising target. Wee1 inhibition, especially when combined with conventional chemotherapy, strongly enhances cell death, at least in part by promoting premature mitosis before DNA replication can be completed during S phase [4].

The identification of Chk1 and Wee1 as targets to enhance replicative stress raises the question whether these kinases communicate with each other, and whether they affect each other's levels and activity. Current knowledge regarding this question is limited. At least in yeast cells [5] and in Xenopus oocyte extracts [6], it has long been known that Chk1 phosphorylates and activates Wee1. But what mechanisms, if any, would allow Wee1 to provide feedback on Chk1 activity?

Saini et al. from the Dobbelstein lab elucidated this by dissecting a signaling chain reaching from Wee1 to Chk1 [7, in press]. We firstly induced replicative stress in our experimental systems using the nucleoside analogue gemcitabine. Under these conditions, we eliminated the activity of Wee1 and its downstream effectors by pharmacological inhibition or by siRNA. As a result, we found that Wee1 inhibition indirectly reduces Chk1 activity as well, by activating cyclin dependent kinases and subsequently suppressing the functions of ATR and the Chk1-coactivator Claspin.

Thus, Wee1 inhibition not only enables premature and thus catastrophic mitosis but also enhances replicative stress by lowering the amount of active Chk1. This may, at least in part, explain the particularly effective synergism between Wee1 inhibitors and chemotherapeutics [4] and encourage the clinical evaluation of Wee1 inhibitors in combination with conventional cancer treatment. Indeed, the Wee1 inhibitor MK-1775 is currently in clinical testing (17 entries in http://clinicaltrials.gov at present).

**Figure:**
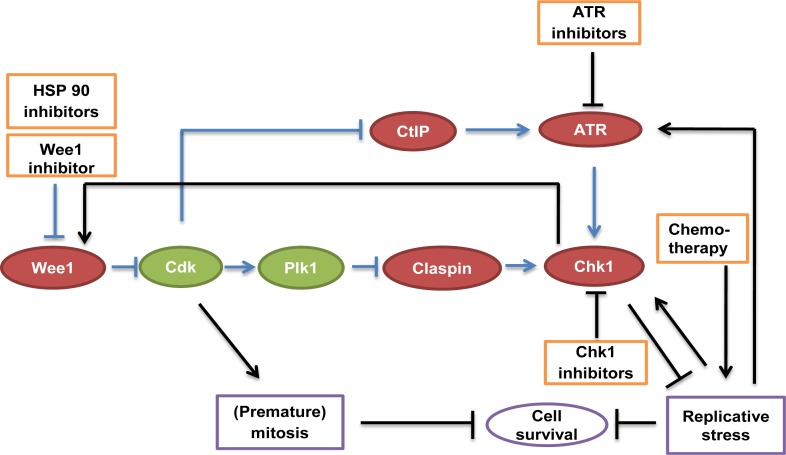
Molecular communication between Wee1 and Chk1 (adapted and expanded from [7]) Wee1 inhibition can be achieved by direct small molecule inhibitors, or otherwise by HSP90 inhibition [9]. Chk1 and ATR are subject to similar inhibition strategies. Kinase activities mediate signaling cross-talk as depicted. Blue arrows indicate pathways investigated in the new study [7], black arrows refer to available literature. Collectively, the model suggests mutual dependence of Wee1 and Chk1 on each other's activities.

Despite the simultaneous impairment of Chk1 activity by Wee1 inhibitors, combining inhibitors of Wee1 and Chk1 may nonetheless prove useful to eliminate cancer cells. This is anticipated since the negative regulation of Chk1 in response to Wee1 inhibition occurs only at a delay of up to 24 hours [7]. Multiple additional drug combinations are conceivable in order to enhance replicative stress [1]. Also, inhibitors of DNA replication, such as nucleoside or base analogues, can be employed to enhance different regimens of cancer treatment. For instance, the Dobbelstein lab has recently observed that the base analogue 5-fluorouracil interferes with homologous recombination repair; it thus augments the damage induced by double strand DNA breaks, as they occur through ionizing irradiation [8].

Exploiting replicative stress for cancer treatment is a strategy that can strongly benefit from the combination of conventional DNA-damaging cancer drugs with targeted signaling inhibitors. Knowing about the cross-talks between these signaling components has the potential of further improving this approach and to identify tumor-cell associated markers for optimizing therapeutic combinations.
